# The Antimicrobial Effect of a Low-Frequency Square Wave Compared to Chlorhexidine

**DOI:** 10.3390/jcm14072429

**Published:** 2025-04-02

**Authors:** Jin-Won Choi, Seon-Mi Byeon, Da-Hyun Lee, Pil-Young Yun, Jeong-Kui Ku

**Affiliations:** 1 Department of Oral and Maxillofacial Surgery, Section of Dentistry, Seoul National University Bundang Hospital, Seongnam 13620, Republic of Korea; jinwon7871@naver.com (J.-W.C.); pilyoung@snubh.org (P.-Y.Y.); 2 Department of Dental Biomaterials, Institute of Biodegradable Materials and Oral Bioscience, School of Dentistry, Jeonbuk National University, Jeonju 54907, Republic of Korea; sumse1205@naver.com; 3 Department of Dental Hygiene, Kyungbuk College, Yeongju 36133, Republic of Korea; ldh16@kbc.ac.kr; 4 Department of Dentistry and Dental Research Institute, School of Dentistry, Seoul National University, Seoul 03080, Republic of Korea

**Keywords:** biofilms, chlorhexidine, extremely low frequency electromagnetic fields, oral hygiene, Schumann resonance

## Abstract

**Background/Objectives**: Oral health is critical for overall health, particularly in hospitalized patients whose weakened physical state can lead to oral changes, such as dry mouth and gingivitis due to anxiety and stress. Neglected oral hygiene can lead to infections and systemic complications. This study aims to evaluate the antibacterial efficacy of low-frequency square-wave positive voltage electrical stimulation compared to chlorhexidine and to assess its potential as a next-generation solution for preventing hospital-acquired infections. **Methods**: Sixty-three tooth specimens were randomly assigned to seven groups, including various concentrations of chlorhexidine and electrical stimulation with or without brushing. Biofilm formation was induced using saliva from healthy donors and standard strains of Streptococcus mutans and Aggregatibactor actinomycetemcomitans. Bacterial colony-forming units (CFU) and absorbance changes were measured post-treatment. **Results**: Significant reductions in CFU counts were observed in both the chlorhexidine and electrical stimulation groups compared to the control, with the 5V2H group showing superior antibacterial efficacy over 0.12% chlorhexidine. Chlorhexidine-treated specimens demonstrated a dose-dependent response and minimal bacterial presence, while electrical stimulation showed effectiveness but with re-growth observed after 4 h. Scanning electron microscopy revealed substantial biofilm on untreated and electrically stimulated specimens, whereas chlorhexidine-treated specimens exhibited minimal bacterial presence. **Conclusions**: Intermittent electrical stimulation shows promise as an alternative to chlorhexidine for oral hygiene management in critical care settings, though an optimization of electrical parameters is necessary for sustained effects. This approach could reduce hospital-acquired infections by providing an effective, non-chemical method for maintaining oral hygiene.

## 1. Introduction

Oral health is a critical factor in maintaining overall health, well-being, and quality of life. Recent studies have identified associations between the oral microbiome and various systemic diseases, including gastrointestinal disorders, cardiovascular diseases, diabetes, and rheumatoid arthritis [1]. Hospitalized patients, particularly those in weakened physical states, can experience oral changes, such as dry mouth and gingivitis due to anxiety and stress. Despite the significant impact of oral hygiene on patients’ systemic health, most hospitalized patients do not receive professional evaluation and management of their basic oral health status within the healthcare delivery system [2,3]. A 2018 study reported that 97.1% of ICU patients required invasive dental treatments, with most having poor biofilm control [4]. Poor oral hygiene promotes the accumulation of dental plaque and the proliferation of pathogenic bacteria, facilitating the spread of pathogens. Endotracheal intubation, in particular, serves as a pathway for oral microorganisms to reach the lungs, increasing the risk of ventilator-associated pneumonia (VAP) [5]. VAP is the second most common infection in ICUs [6], with a mortality rate of up to 13% [7]. Efficient oral hygiene management is essential in reducing the incidence of pneumonia, as has been indicated by multiple studies [8,9,10,11].

Currently, oral care predominantly involves the use of chlorhexidine antiseptics [12]. However, the long-term use of chlorhexidine can cause adverse effects, such as tooth discoloration, taste alteration, and antimicrobial resistance [13,14]. Consequently, there is a demand for effective and sustainable alternatives to traditional oral disinfection methods. Research on alternative oral disinfection methods, including the use of ions and ultrasonic waves, is increasing [15,16,17], with a substantial focus being on the potential of electrostimulation. Bacterial life, growth, and function depend on the bioelectrical environment, which, when disrupted, impairs biofilm formation and inhibits bacterial proliferation [18]. Several studies have reported that electrostimulation inhibits bacterial growth through direct and indirect mechanisms [19,20,21]. The natural electromagnetic resonance known as the Schumann frequency, occurring at 7.83 Hz due to vibrations between the Earth’s surface and ionosphere, has shown cell growth inhibitory effects and has been observed to inhibit actinomyces bacteria for over six hours [22,23,24].

However, existing studies have primarily focused on exploring the standalone effects of electrostimulation or its synergistic effects when combined with antimicrobial agents, with few comparing the antibacterial effects of electrostimulation alone versus chlorhexidine alone. Therefore, this study aims to compare the antibacterial efficacy of low-frequency square wave positive voltage electrostimulation with chlorhexidine and explore its potential application as a next-generation solution for preventing hospital-acquired infections.

## 2. Materials and Methods

### 2.1. Preparation of Tooth Specimens

Healthy incisors without defects such as cracks or white spots were used. Using a low-speed handpiece, tooth specimens were cut into cylindrical shapes with a diameter of 7 mm and height of 3 mm. The cut specimens were sequentially polished with 600, 800, 1000, and 1200 grit sandpaper while cooling with water. A total of 63 tooth specimens were prepared, with 9 specimens per group.

### 2.2. Experimental Groups

The specimens were randomly assigned to seven groups based on the treatment method (Table 1).

#### 2.2.1. Chemical Antimicrobial Agents

To evaluate the effects of chemical antimicrobial agents on biofilm, specimens were immersed in 0.12% and 0.5% chlorhexidine solutions for 30 s.

#### 2.2.2. Electric Stimulation

**Figure 1:**
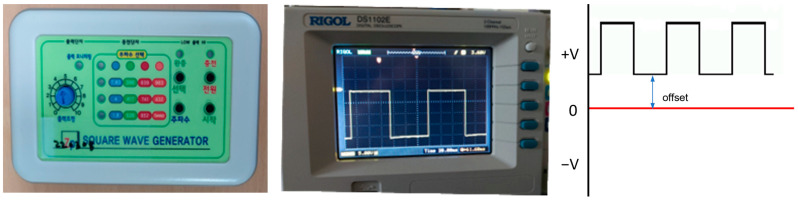
A square wave generator with a continuous positive voltage offset of 0.7 V.

A square wave generator (SWG) capable of applying low-frequency square wave positive voltages below 1000 Hz, which had been previously developed, was used (Figure 1). The generator was designed to apply specific voltage and frequency settings to assess the effect of electric stimulation on biofilm. Based on previous research, the most effective conditions for inhibiting *A. actinomycetemcomitans* growth were determined [22]. The experimental conditions were set accordingly, with a continuous positive voltage offset of 0.7 V and a low-frequency square wave AC of 7.83 Hz at 5 V applied to the specimens for 2 h.

#### 2.2.3. Brushing

To evaluate the combined effect of chemical antimicrobial agents or electric stimulation and physical cleaning, a brushing protocol involving five strokes was added after the chemical antimicrobial agent or electric stimulation treatment. Brushing was performed consistently across all specimens to ensure uniformity.

### 2.3. Biofilm Formation

#### 2.3.1. Saliva Collection

Saliva donors were healthy adults with no history of antibiotic use in the past three months, no caries or periodontitis, and overall systemic health. Donors refrained from any oral hygiene activities for 24 h prior to saliva collection. Collected saliva was filtered using sterile glass wool to remove impurities.

#### 2.3.2. Biofilm Culture

Tooth specimens were immersed in the donor’s saliva for 6 days, with 0.1 ml of 0.2% sucrose added daily. After saliva removal, specimens were cultured with *S. mutans* and *A. actinomycetemcomitans* for biofilm formation, applied in a fresh culture medium (basal medium mucin growth medium mixed with 0.3% sucrose) and incubated at 37 °C in a new medium well every 24 h for a total of 7 days [25].

#### 2.3.3. Standard Strains

The standard strains used were Streptococcus mutans and *Aggregatibacter actinomycetemcomitans* KCOM1304, provided by the Korean Oral Microbiome Resource Bank. Strains were cultured in Brain Heart Infusion (BHI) broth at 37 °C in a 5% CO_2_ incubator for 24 h, then adjusted to a density of 1.5 × 10^8^ using a DensiCHEK Plus turbidometer(SKU 21255, bioMerieux, Cambridge, MA, USA) for the experiments.

### 2.4. Colony-Forming Unit (CFU) Measurement

Post-treatment, bacterial colonies were evaluated by measuring colony-forming units. Treated tooth specimens were immersed in cysteine peptone water (CPW), and 100 µL of the bacterial solution was spread onto solid media (BAP) for 24 h before colony counting. CFU/mL was calculated using the formula:

$$\mathrm{Colony}-\mathrm{Forming}\;\mathrm{Unit}\;\left(\mathrm{CFU}/\mathrm{mL}\right)=\;\mathrm{Number}\;\mathrm{of}\;\mathrm{colonies}\times 1/\mathrm{dilution}\;\mathrm{factor}\;\times 1/\mathrm{inoculum}\;\mathrm{volume}\;\left(\mathrm{mL}\right)$$

### 2.5. Absorbance Measurement

The persistence of bacterial growth inhibition over time was evaluated by measuring changes in absorbance. Treated tooth specimens were placed in CPW solutions, and 200 µL of the bacterial solution was loaded into 96-well plates. Absorbance at 600 nm was measured using an ELISA reader (Precision microplate reader, Molecular Devices, San Jose, CA, USA) over time.

### 2.6. High-Resolution Field Emission Scanning Electron Microscopy (HR FE-SEM) Observation

To observe biofilm formation on the tooth specimens, they were washed three times with 1 mm CPW to remove loose bacteria. Specimens were then placed in conical tubes containing 2 mL of CPW and subjected to vortexing and sonication for one minute to disperse the biofilm. HR FE-SEM was used to observe the specimen surfaces at magnifications of 1000× and 5000×. Additionally, bacteria detached after chlorhexidine or electric stimulation treatment were fixed and observed at 3000× magnification.

### 2.7. Statistical Analysis

Data were tested for normality using the Kolmogorov–Smirnov test and analyzed for statistical significance using one-way ANOVA. Tukey’s post hoc test was applied to compare differences between groups.

## 3. Results

### 3.1. Bacterial Colony Counts

Table 2 shows the bacterial colony counts post-treatment. Both chlorhexidine and electric stimulation groups showed significant reductions in bacterial colonies compared to the control group (*p* < 0.05). Chlorhexidine groups demonstrated a dose-dependent response, with higher concentrations showing more pronounced antibacterial effects (*p* < 0.05).

The addition of brushing further inhibited bacterial colony formation in both chlorhexidine and electric stimulation groups. The CHX0.12B group had fewer bacterial colonies than the CHX0.12 group, and the CHX0.5B group had fewer colonies than the CHX0.5 group. The CHX0.12B group also had fewer colonies than the CHX0.5 group (*p* < 0.05).

**Figure 2:**
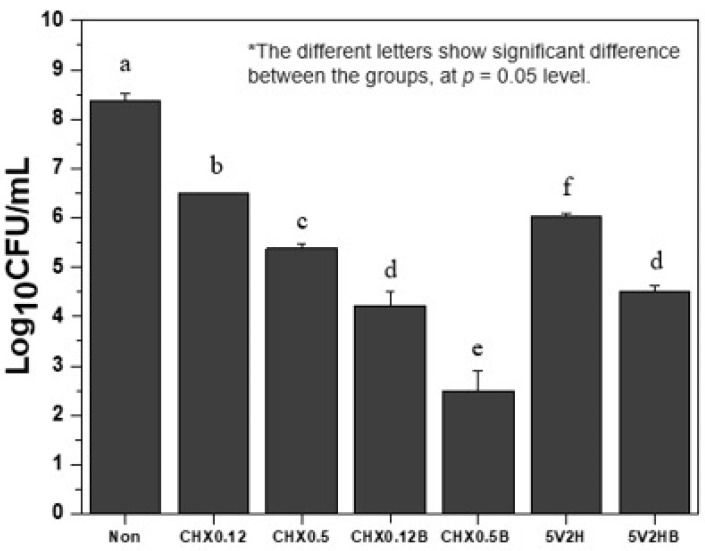
Bacterial CFU counts after treatment.

In comparing chlorhexidine and electric stimulation, the 5V2H group had fewer bacterial colonies than the CHX0.12 group but more than the CHX0.5 group (*p* < 0.05). The 5V2HB group had slightly more colonies than the CHX0.12B group, but the difference was not statistically significant (*p* > 0.05). The 5V2HB group had fewer colonies than the CHX0.5 group (*p* < 0.05) (Figure 2).

### 3.2. Absorbance Changes

**Figure 3:**
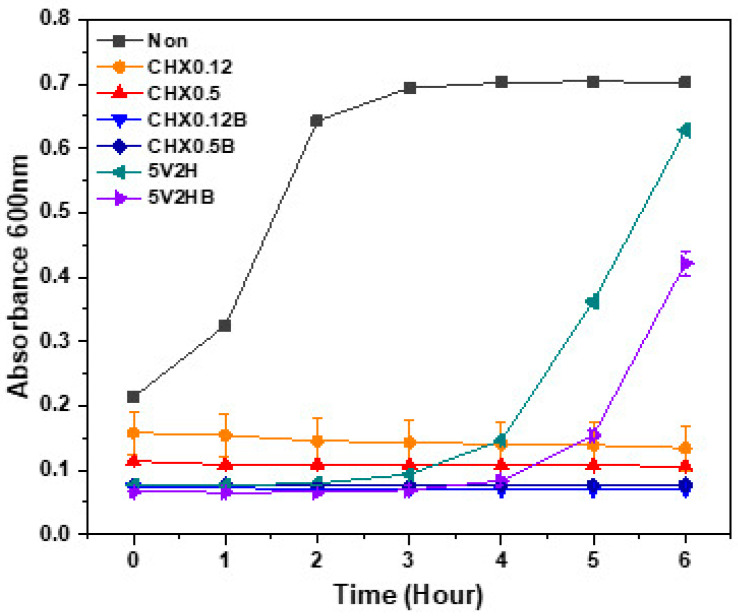
Bacterial growth curve after treatment.

Table 3 and Figure 3 show the changes in absorbance at 600 nm over time post-treatment. In the untreated control group, absorbance increased from the start of incubation, with a sharp rise observed between one and two hours, followed by a gradual increase. In contrast, chlorhexidine groups showed no significant changes in absorbance over six hours, regardless of concentration or brushing. However, the 5V2H group showed no change in absorbance for the first three hours, followed by an increase after four hours. The 5V2HB group showed a delayed increase in absorbance, maintaining a stable level for the first four hours before rising.

### 3.3. Scanning Electron Microscopy (SEM) Observation

**Figure 4:**
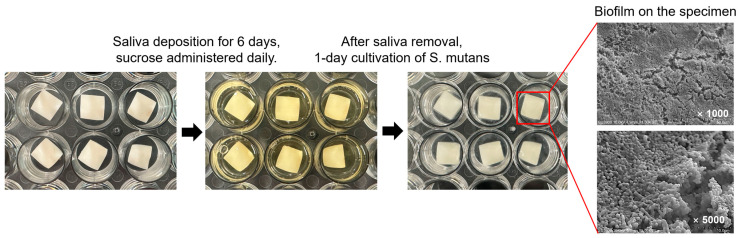
Biofilm formation process on the specimen.

**Figure 5:**
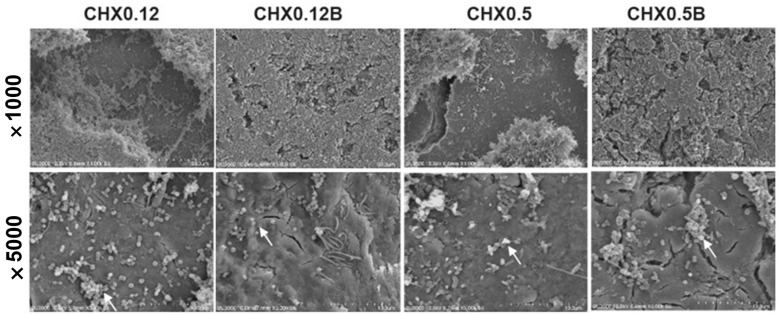
SEM image of the biofilm on the specimen after chlorhexidine treatment. The CHX0.5 group shows a greater reduction in bacterial colonies compared to the CHX0.12 group, and the group that underwent brushing shows biofilm removal with bacterial deformation observed (arrow). (CHX0.12: Immersion in 0.12% chlorhexidine; CHX0.12B: Immersion in 0.12% chlorhexidine and brushing; CHX0.5: Immersion in 0.5% chlorhexidine; CHX0.5B: Immersion in 0.12% chlorhexidine and brushing).

Untreated control specimens were uniformly covered with biofilm, including *S. mutans* and *A. actinomycetemcomitans* (Figure 4). Chlorhexidine-treated specimens showed a significant reduction in biofilm, with the CHX0.12B group showing minimal biofilm and bacterial deformation at 5000× magnification (Figure 5). The CHX0.5 group showed a greater reduction in bacterial colonies compared to the CHX0.12 group, with brushing further reducing biofilm presence and deformation observed at 5000× magnification (Figure 5).

**Figure 6:**
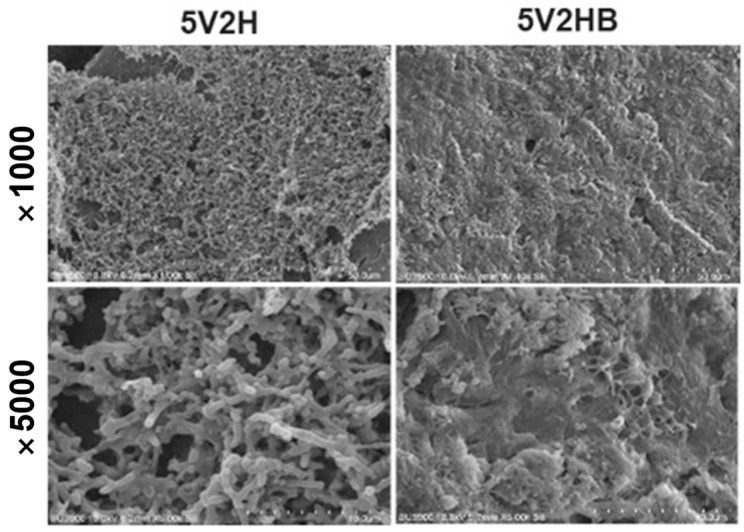
SEM image of biofilm on the specimen after electric stimulation. Partial bacteria were removed. (5V2H: A low-frequency square wave AC at 7.83Hz was applied to the specimen at 5V for 2 h.; 5V2HB: 5V2H with brushing).

In the 5V2H group, biofilm covered the tooth surface similar to that of the control group, and the 5V2HB group showed only partial biofilm removal with lower layers remaining intact (Figure 6).

**Figure 7:**
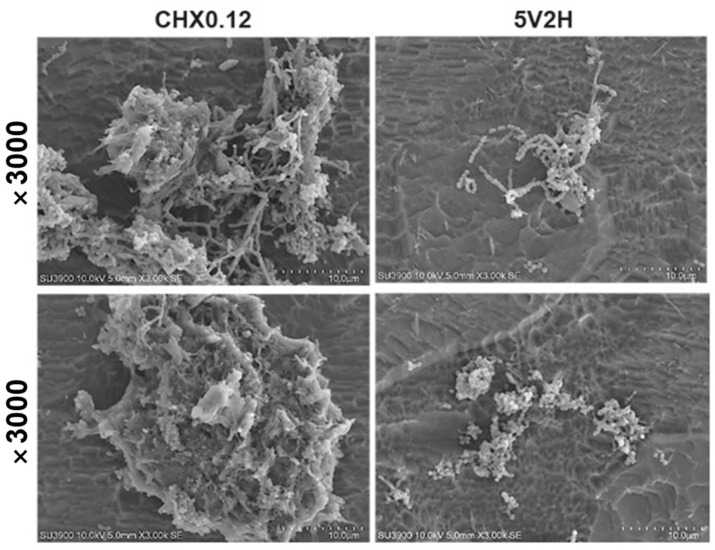
SEM image of bacteria detached from specimen after treatment. In the CHX0.12 group, the bacteria were observed to detach in clusters, whereas in the 5V2H group, they detached individually or only parts of the biofilm were detached. (CHX0.12: Immersion in 0.12% chlorhexidine; 5V2H: A low-frequency square wave AC at 7.83 Hz was applied to the specimen at 5 V for 2 h).

Detached bacteria were observed in clusters in the CHX0.12 group, whereas in the 5V2H group, bacteria detached individually or only parts of the biofilm were removed (Figure 7).

## 4. Discussion

Electrical stimulation has been applied in various medical fields due to its additional effects of promoting tissue regeneration and increasing blood flow [26,27,28]. Since Rowley first reported the antibacterial effects of electrical stimulation in 1972 [29], it has been shown to induce direct bacterial death by disrupting bacterial cell membrane integrity or electrolyzing bacterial surface molecules. Indirectly, electrical stimulation induces pH changes and produces toxic substances such as hydrogen peroxide through electrolysis, further inhibiting bacterial growth [19]. These antibacterial effects of electrical stimulation have reinforced the concept of its innovative application in medicine. This study evaluates the potential application of electrical stimulation as a next-generation solution for preventing oral infections in ICU patients by comparing its antibacterial efficacy with that of chlorhexidine. The antibacterial effect of low-frequency square wave positive voltage was found to be comparable to chlorhexidine treatment combined with brushing, maintaining colony formation inhibition for at least four hours. Electrical stimulation showed a biofilm-disrupting pattern, suggesting its potential as an oral hygiene management device for hospitalized patients where biofilm control is crucial.

Generally, oral care in hospitals involves chlorhexidine according to nursing guidelines, but it carries risks such as tooth discoloration, taste alteration, and an increased risk of aspiration pneumonia [3]. Although electrical stimulation did not surpass the antibacterial effect of 0.5% chlorhexidine alone, it had a comparable effect for 4 h to the commonly used 0.12% chlorhexidine. Therefore, intermittent electrical stimulation could be an effective antibacterial alternative to chlorhexidine in clinical settings. A 2024 study observed a reduction in dental plaque bacteria using high-frequency electrical stimulation, consistent with our findings [30]. Adding brushing to electrical stimulation achieved similar antibacterial effects to the 0.12% chlorhexidine and brushing group, and better results than 0.5% chlorhexidine alone. This synergy suggests a shift towards combining traditional brushing with innovative treatments like electrical stimulation [31]. Multiple studies have reported that electrical stimulation alters the biofilm structure of bacteria, enhancing the effectiveness of antibacterial agents [32,33,34]. Using both traditional antibacterial agents and electrical stimulation may be more effective in inhibiting oral biofilm formation

Using absorbance changes, the persistence of the antibacterial effects of electrical stimulation and chlorhexidine was evaluated. Chlorhexidine showed sustained antibacterial effects over the six-hour observation period, regardless of brushing. Chlorhexidine’s cationic properties allow it to bind to the negatively charged tooth surface and salivary glycoproteins, providing excellent surface adhesion and up to 12 h of antibacterial efficacy [35]. Conversely, the antibacterial effect of electrical stimulation decreased after four hours, leading to subsequent bacterial regrowth (Figure 3). SEM observations revealed significant differences in biofilm presence on the tooth specimen surfaces (Figure 4, Figure 5, Figure 6 and Figure 7). Chlorhexidine-treated specimens showed bacterial removal in clusters, while electrical treatment resulted in biofilm decomposition and bacterial removal. This difference could be attributed to chlorhexidine’s mechanism of action, which directly targets bacterial cells, adhering to and disrupting cell walls and internal functions, coagulating cytoplasmic contents, and causing bacterial death [36]. In contrast, electrical stimulation relies on localized effects like membrane and cell wall damage or protein synthesis inhibition, limiting its antibacterial effects to directly applied areas [19,37,38]. Therefore, to sustain the antibacterial effects of electrical stimulation, its intermittent application every 4–5 h is necessary, and the development of devices to apply electrical stimulation to necessary areas is required.

This study used specific size, shape, and time parameters of positive square wave voltages identified in previous research as most effective against *A. actinomycetemcomitans* [22]. Square wave voltages can maintain magnetic field strength consistently and exhibit significantly lower cell survival rates than sine waves [39]. The application time was based on various studies indicating that longer stimulation decreases bacterial growth rates [40,41]. However, this specificity of parameters may limit the exploration of different antibacterial effects under varying voltages, frequencies, waveforms, and application times. While this study using low-frequency electrical stimulation showed specimens mostly covered in bacteria under SEM, another study using high-frequency showed contrasting results [30]. These discrepancies highlight the importance of specific electrical stimulation parameters in achieving effective antibacterial outcomes. Future research should focus on adjusting and optimizing parameters like voltage, frequency, waveform, and time to enhance the antibacterial efficacy of electrical stimulation, broadening its antibacterial range and duration.

This study has several limitations. Firstly, the diversity of biofilms is limited. This study used specific bacterial strains significant in oral infections like periodontitis and caries [42,43]. However, oral biofilms are complex and composed of multiple bacterial species, and the study results may not apply to the diverse microbial environments found in clinical settings. Secondly, as an in vitro study, it did not account for individual differences in saliva composition, immune responses, and other oral conditions that could affect outcomes. Lastly, the short observation period limits insights into potential bacterial regrowth and resistance development, and the study did not evaluate potential side effects related to electrical stimulation. Future research should include various comparison groups, long-term longitudinal studies using multispecies biofilm models, and in vivo studies to confirm clinical applicability and effectiveness. To advance this technology into a medical device suitable for clinical use, it is essential to design the device with careful consideration of the anatomical characteristics of the application site and the placement of the electrodes. Additionally, its usability, suitability, safety, and potential risks to the human body must be verified through comprehensive preclinical and clinical studies. This direction will explore the practical application of research findings in clinical settings and provide crucial guidelines for future treatment development.

## 5. Conclusions

Low-frequency square wave electrical stimulation demonstrated comparable antibacterial effects to chlorhexidine but did not completely eliminate bacteria, with regrowth observed after four hours. Intermittent electrical stimulation devices could be developed for use in patients with limited oral care abilities, potentially preventing infections effectively. Future research should focus on optimizing electrical stimulation parameters and conducting in vivo studies to confirm these findings in clinical settings.

## Figures and Tables

**Table 1:** Experimental groups.

Group	
Non	Untreated
CHX0.12	Immerse in 0.12% chlorhexidine solution for 30 s
CHX0.12B	Immerse in 0.12% chlorhexidine solution for 30 s + Brushing 5 times
CHX0.5	Immerse in 0.5% chlorhexidine solution for 30 s
CHX0.5B	Immerse in 0.5% chlorhexidine solution for 30 s + Brushing 5 times
5V2H	Electrotherapy (Voltage of 5 V, for 2 h)
5V2HB	Electrotherapy (Voltage of 5 V, for 2 h) + Brushing 5 times

**Table 2:** Bacterial CFU counts after treatment.

Group	AVERAGE	SD
Non	8.35758	0.16905
CHX0.12	6.49114	0.01982
CHX0.5	5.37409	0.10333
CHX0.12B	4.21568	0.30502
CHX0.5B	2.5	0.4
5V2H	6.03959	0.05599
5V2HB	4.50515	0.13705

**Table 3:** Absorbance changes according to bacterial culture time after treatment.

Time (Hours)	Non		CHX0.12		CHX0.5		CHX0.12B		CHX0.5B		5V2H		5V2HB	
	Average	SD	Average	SD	Average	SD	Average	SD	Average	SD	Average	SD	Average	SD
0	0.21367	0.00208	0.15833	0.03361	0.11433	0.00057	0.07333	0.00057	0.07667	0.00055	0.077	0.0001	0.06667	0.00057
1	0.32467	0.00751	0.15433	0.03296	0.10867	0.00115	0.073	0	0.07733	0.00208	0.077	0.002	0.06567	0.00053
2	0.643	0.004	0.14567	0.03424	0.109	0.00173	0.07067	0.00057	0.076	0.00265	0.07967	0.00208	0.066	0.0001
3	0.694	0.00458	0.143	0.03559	0.108	0.00173	0.071	0.0001	0.07633	0.00416	0.09367	0.00208	0.06933	0.00115
4	0.702	0.00361	0.14033	0.03512	0.10733	0.00153	0.07	0	0.07667	0.00473	0.14667	0.00321	0.08367	0.00153
5	0.70433	0.00493	0.139	0.03451	0.107	0.00173	0.06967	0.00053	0.07733	0.00586	0.36233	0.00513	0.15467	0.00666
6	0.702	0.002	0.13467	0.03508	0.10667	0.00115	0.06967	0.00054	0.07667	0.00643	0.62833	0.00153	0.42133	0.0179

## Data Availability

All data generated or analyzed are included in this article. Further inquiries can be directed to the corresponding author.
